# Education in focus: Significant improvements in student learning and satisfaction with ophthalmology teaching delivered using a blended learning approach

**DOI:** 10.1371/journal.pone.0305755

**Published:** 2024-07-01

**Authors:** Andrea J. Doyle, Conor C. Murphy, Fiona Boland, Teresa Pawlikowska, Joan Ní Gabhann-Dromgoole

**Affiliations:** 1 SIM Centre for Simulation Education and Research, RCSI, University of Medicine and Health Sciences, Dublin, Ireland; 2 Dept of Ophthalmology, Royal Victoria Eye and Ear Hospital (RVEEH), RCSI, University of Medicine and Health Sciences, Dublin 2, Ireland; 3 Data Science Centre, RCSI, University of Medicine and Health Sciences, Dublin, Ireland; 4 Health Professions Education Centre (HPEC), RCSI, University of Medicine and Health Sciences, Dublin, Ireland; 5 School of Pharmacy and Biomolecular Sciences, RCSI, University of Medicine and Health Sciences, Dublin 2, Ireland; University of Lisbon: Universidade de Lisboa, PORTUGAL

## Abstract

**Purpose:**

This study aimed to measure student satisfaction with a revised ophthalmology delivery format, which due to the pandemic had previously relied on a remote online flipped classroom (OFC) format compared to a blended learning format. This educational strategy combined online learning with in-person seminars and practical patient centred sessions. Our previous investigations demonstrated a significant lack of student satisfaction with a curriculum solely reliant on a remote OFC, as such we hypothesised that a blended learning approach would result in improved levels of student satisfaction and knowledge gain.

**Methods:**

Non-randomised intervention study of two groups; group 1 = OFC group and group 2 = BL group, compared perspectives of 4th year ophthalmology students using a validated course evaluation questionnaire (CEQ).

**Results:**

A total of 59 students from the BL group (n = 257; response rate = 23.0%) and 28 from the OFC group agreed to participate in the study (n = 114; response rate = 24.6%). Participants in the BL group felt it was easier to determine the standard of work that was expected (77.42% v 60.71%) and demonstrated significantly increased satisfaction with staff motivation of students (95.16% v 64.29%, p <0.001) and provision of feedback (74.19% v 46.43%, p = 0.004), compared to the OFC group. Furthermore, students in the BL group also felt the course significantly improved their analytical skills (64.52% v 42.85%, p = 0.023) and ability to work as part of team (69.36% v 25%, p <0.001) as well as reporting reduced dissatisfaction with the level of choice afforded in terms of how they would learn (33.88% v 60.71%, p = 0.31) and the how they were assessed (59.68% v 89.28%, p = 0.004). No evidence of a statistical difference in exam score was observed.

**Conclusions:**

The COVID-19 pandemic necessitated an unavoidable pivot to online and distance learning, to meet the challenges presented by government mandates and social distancing requirements. Since many of these directives have been reversed, it is important to evaluate the effectiveness and learner perceptions’ of the online and distance learning interventions. In this study we demonstrated a significant student preference for BL compared to the OFC approach, with comparable student performances determined by MCQ examinations. Our findings suggest a preference for reintroducing in-person and patient engagement activities in post-pandemic health professions education.

## Introduction

The necessities brought about by the COVID-19 pandemic required an inevitable shift towards online and distance learning, addressing the challenges posed by government directives, the need for social distancing, and continuing health professions education (HPE) [1–4]. Reviews that investigated the developments in medical education in response to the COVID-19 pandemic highlighted that in the immediate response, the majority of interventions described a pivot to online learning. However, the need for continued clinical contact remained but was often replaced in curricula with remote, distance or telehealth. In the Best Evidence in Medical Education (BEME) rapid review, BEME 63, the authors identified a significant focus on sharing experiences, rather than robust evaluation or research enquiry, and less than 50% of studies reviewed described educational outcomes. BEME Guide no.64 acknowledged that online learning will undoubtedly continue to be a feature of medical education long after the pandemic, but encouraged educators to have a deliberate and thoughtful selection of strategies and consider the differential impacts of these approaches. BEME Guide no. 71 recognised the limitations of remote learning including the loss of social interactions, lack of hands-on experiences and challenges with technology, the authors recommend its continued use in higher education due the flexibility it offered and highlighted practical advice to optimize the online environment [4]. Among the educational interventions that were adopted in response to the COVID-19 pandemic, the flipped classroom (FC) has been reported to be efficacious in responding to these extraordinary challenges in medical education [5–7]. The FC, a form of blended learning and instructional strategy, seeks to enhance student engagement and learning. It involves students completing readings autonomously outside of scheduled class time and participating in live problem-solving activities during class time. In undergraduate ophthalmology education, studies by Diel *et al* found high levels of satisfaction with a FC approach and reported no changes in knowledge acquisition [8], and a reduction in students’ pressure to perform, course burden and anxiety, along with increased confidence in triaging common eye complaints [9].

In our initial educational response to the pandemic at our university, we implemented a remote online flipped classroom (OFC) approach to facilitate delivery of an ophthalmology clinical attachment for medical students, and evaluated students perceptions and satisfaction with the Course Evaluation Questionnaire (CEQ) [10]. The CEQ is used globally to determine undergraduate student satisfaction and to identify areas for improvement [11, 12]. There is substantial evidence supporting its reliability and validity with undergraduate and medical students [13–19], and it has been utilised in ophthalmology interventions evaluating the FC [20–22]. However, the efficacy of the FC for ophthalmology education in a completely virtual setting is still insufficiently measured [9]. We investigated student satisfaction using the CEQ following the introduction of a remote online FC, necessitated by the COVID-19 pandemic, compared to with our usual delivery format, which provided a blend of didactic lectures and clinical skills sessions [10].

Our results contradicted existing literature on the effectiveness of a flipped-classroom approach in delivering ophthalmology content to medical students. Previous studies indicated a preference among students for the flipped classroom over the traditional lecture method, citing its benefits in developing problem-solving, creative thinking, and teamwork skills [21, 23]. We identified significant levels of dissatisfaction with problem solving, communication, staff motivation and provision of feedback [10]. As the constraints imposed by government directives and the necessity for social distancing eased, we sought to re-design the ophthalmology module to incorporate learnings from our previous findings and the evidence based recommendations from BEME reviews 63, 64, 69 and 71 [1–4]. For subsequent iterations of the ophthalmology module an educational strategy that combined online learning and in-person seminars with practical patient-centred sessions was adopted. It was anticipated that this blended learning approach would result in improved levels of student satisfaction and knowledge gain. In this study we investigated how a blend of traditional classroom-based and remote FC learning approaches combined with in person practical elements including direct patient contact would impact student satisfaction with the CEQ, and compare these results to those previously reported for the complete OFC delivery of ophthalmology content [10].

## Methods

### Study populations

Participants in this study were 4^th^ year senior cycle medical students enrolled in RCSI on an Ophthalmology clinical attachment that takes place 20 times during the academic year. All students undertaking the ophthalmology clinical attachment module were invited to participate. This study was reviewed and approved by the Research and Ethics Committee (REC) of the RCSI, University of Medicine and Health Sciences and was conducted according to the principles expressed in the Declaration of Helsinki. Written informed consent was obtained from all participants (REC 202006015).

#### Group 1: Online flipped classroom (OFC) group (2019/2020 ophthalmology module)

As a result of the global pandemic an online distance module was devised for students participating in the ophthalmology clinical attachment for the 2019/2020 academic year. As previously described these students (total n = 114) engaged in a curriculum solely dependent on an online flipped classroom (OFC) and for the purposes of the current study functioned as our comparison group [10]. Recruitment period for this study cohort: 19^th^ October 2020 to 18^th^ December 2020.

#### Group 2: Blended Learning (BL) group (2020/2021 ophthalmology module)

The Blended learning (BL) of the 4^th^ year ophthalmology clinical attachment began on the 5^th^ of October 2020 in the Royal Victoria Eye and Ear Hospital (RVEEH) and finished 26^th^ April 2021. Recruitment period for this study cohort: 5^th^ April 2021 to 28^th^ May 2021. Students were assigned into groups by the SARA (Student, Academic & Regulatory Affairs) office RCSI (10–12 in each) before commencing their clinical attachment week. BL students attended on site teaching sessions, remote online FC sessions and in-person patient centred clinical skills teaching sessions.

#### Module description

The aims of the ophthalmology module were to enable the students to develop the clinical knowledge and skills to assess any patient presenting with an eye disorder and to formulate an appropriate differential diagnosis and management or referral plan.

Our objective was to ensure constructive alignment of the module with the existing learning outcomes (S1 Appendix) despite the change in delivery whilst responding to the changes precipitated the by COVID-19 pandemic regarding social distancing [24].

- ***On site in-person teaching***: students attended sessions on the Anatomy of the Eye, History taking in ophthalmology, Patient based teaching and Clinical skills, which each consisted of 60-min small group teaching sessions (face to face lectures with 15-minute question and answer session) led by an Ophthalmologist.
- ***Online flipped classroom*:** students were asked to watch pre-recorded video lectures (Cataract, Glaucoma, Diabetic Retinopathy, AMD) online in advance of a one hour interactive session led by an Ophthalmologist. This was supplemented by slide sets of the didactic lecture material without audio. After the pre-class lecture students attended a synchronous, online, live interactive session on the same topic. These Blackboard Collaborate (BBc) sessions included problem solving, clinical vignettes and MCQs relating to the recorded lecture. Additional BBc sessions covered 3 other key topics including red eye, sudden loss of vision and change in appearance. Facilitators prepared clinical cases and related MCQs that addressed learning outcomes and promoted engagement for use during the interactive online session. The facilitator encouraged problem solving using the poll feature of BBc which encouraged both discussion and active learning.
- ***Clinical skills***: students attended in person, practical clinical skills sessions which included the examination of the following: Snellen visual acuity, direct ophthalmoscopy, eye movements, pupil reactions, visual fields to confrontation, cover test for strabismus and the external eye examination with a pen torch.
- ***Patient centred teaching***: students engaged in in-person patient led teaching practical sessions which consisted of taking patient history, reading patient charts, examining patients and a discussion regarding the outcomes of the consultation facilitated by the supervising clinical tutor. Students also attending outpatient clinics as observers, listening to patient histories, examining clinical signs and discussing patient cases with the attending doctors. Knowledge was tested upon completion of the module via a multiple- choice question (MCQ) exam. Clinical Competency (skills) were assessed by practical examination of fundoscopy skills.

### Digital training

Blackboard Collaborate (BBc) has previously been shown to have utility as a platform to support nursing students placement learning. Several studies have highlighted the importance training to develop students digital literacy to facilitate student engagement with this form of technology [25, 26]. To support this guides to the use of BBc were prepared and provided to the students ahead of the online module. Digital training was provided to ophthalmology faculty along with support guides for the use of the BBc platform.

### Instrument and data collection

To investigate student perceptions and satisfaction with the online flipped classroom, all students (257) were invited to complete the CEQ36 online via Survey Monkey. Each item of the questionnaire is answered using a standard 5-point Likert scale where the levels of agreement range from “strongly agree” (scoring a “1”) to “strongly disagree” (scoring a “5”). The CEQ36 measures six constructs established as important learning environment features within the context of higher education [12, 14, 16], and are presented in Table 1. In addition to the CEQ36 data, final anonymised MCQ exam scores were obtained for each student in the study.

**Table 1:** Course Evaluation Questionnaire constructs, dimensions and indicators.

Construct	Dimensions	Related items in CEQ 36
Good Teaching (GT) scale	Assesses the degree to which students felt that the teaching staff provided a high level of teaching quality including adequate feedback, presentation of course content, and whether students felt information was presented in a clear and understandable manner.	Q4, Q9, Q20, Q22, Q23, Q25, Q31, Q33
Clear Goals and Standards (CG) scale	Assesses the degree to which participants felt they had a clear understanding of the standard of work expected and teaching staffs’ expectations of students during the course.	Q1, Q8, Q18, Q24, Q35
Appropriate Assessment (AA) scale	Gauges student’s perceptions of the assessment strategies, specifically if the assessment was focused on memorising information instead of understanding.	Q7, Q10, Q17, Q26, Q29, Q32,
Appropriate Workload (AW) scale	Addresses the student’s perceptions of the workload including volume timing and expectations during the course.	Q5, Q14, Q19, Q27, Q36
Generic Skills (GS) scale	Gauges the extent to which the course adds to the generic skills that students might be expected to possess on completion of the course. These are not discipline specific skills, they are associated with communication, problem solving and teamwork.	Q2, Q6, Q11, Q12, Q13, Q28
Emphasis on Independence (IN) scale	Evaluates student’s perceptions on their autonomy and choice in their participation in their learning and engagement.	Q3, Q15, Q16, Q21, Q30, Q34, Q37

### Statistical analysis

Descriptive statistics were used to describe the characteristics of the two groups (OFC (n = 28) vs BL (n = 59)) and Chi-square test/Fisher exact test, or independent samples t-test used to explore differences between the groups. The scores of the MCQ final exam were compared using independent samples t test. The questionnaire data given to students were analysed using Mann-Whitney-U tests, to explore potential differences between the groups. During analysis responses for the ‘agree’ and ‘strongly agree’ categories were combined, similarly responses for ‘disagree’ and ‘strongly disagree’ category were combined. All statistical analyses were performed in GraphPad Prism V5 or Stata v13.

## Results

A total of 257 undergraduate medical students who received the BL delivery of the ophthalmology clinical attachment were invited to participate in this study. Of these, 59 students (23%) agreed to take part in the study and completed an online CEQ. A total of 114 students who had received OFC delivery of ophthalmology content the year prior attended online tutorials as described previously [10]. Of these, 28 agreed to participate (25%).

The demographic distribution of the participants is presented in Table 2. There was no evidence of a difference in gender or age between the OFC and BL groups for the classes as a whole (column 1 v 3), or between students in the OFC group or the BL group who participated in the online surveys (column 2 v 4).

**Table 2:** Demographic information of students who participated in OFC and BL deliveries.

	Group 1: Online flipped classroom 2019/2020		Group 2: Blended Learning classroom 2020/2021	
	All Students	OFC Survey Respondents	All Students	BL Survey Respondents
Number of students (n)				
	114	28	257	59
Gender (n) %				
Male	53 (45.6%)	13 (46.4%)	99 (38.5%)	16 (27.1%)
Female	61 (53.5%)	15 (53.6%)	158 (61.5%)	43 (72.9%)
Age				
(years old), mean ± SD	26.5±2.8	25.6±2.3	26.6±4.1	26.5±4.6

### Student perceptions

**Fig 1:**
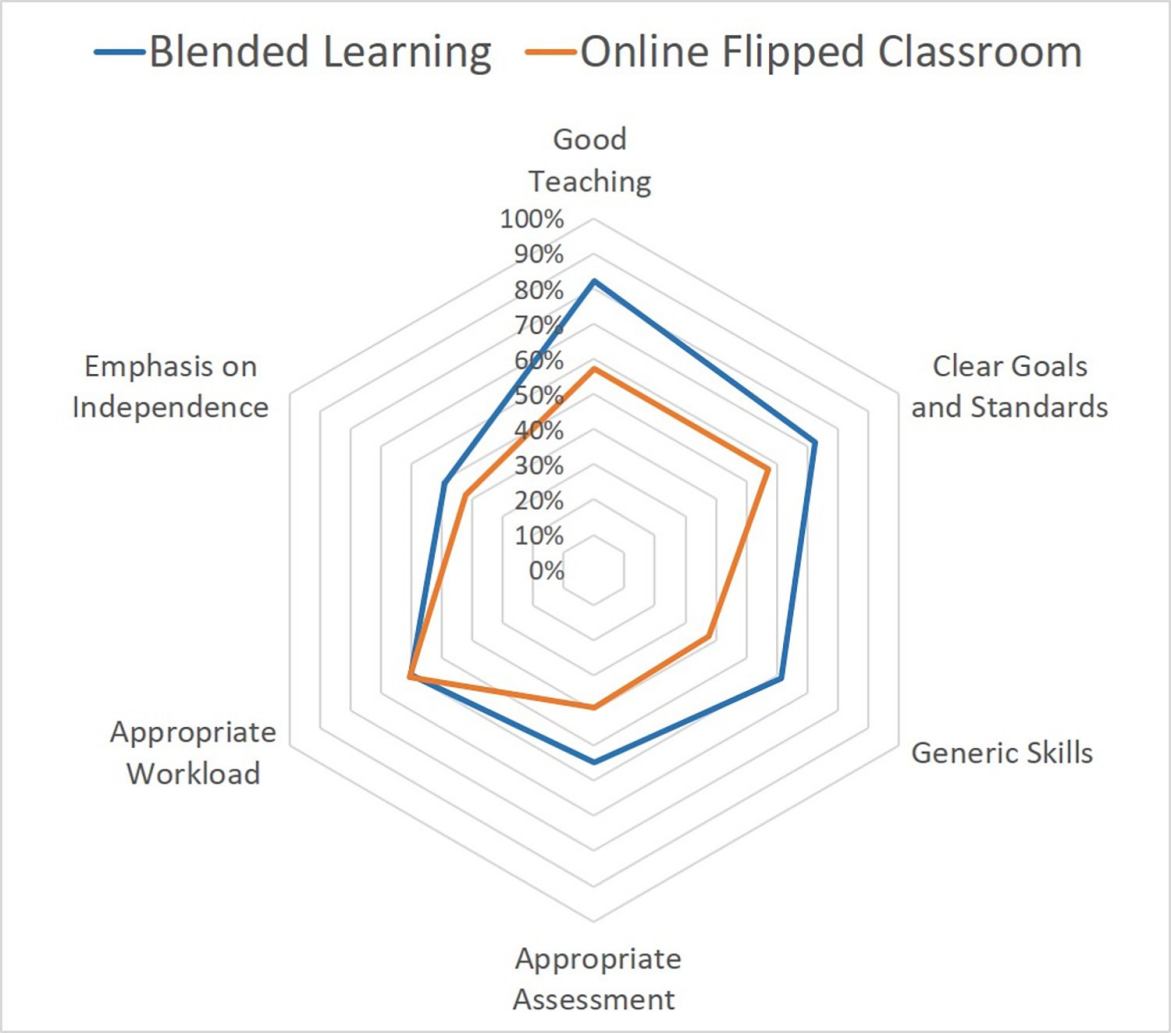
Global summary of CEQ comparing student perceptions within the six survey constructs. Average values were derived from analysis of Likert scale responses to each question within a subscale. OFC = Online flipped classroom. BL = Blended learning.

Fig 1 graphically summarizes the responses from the students regarding the six constructs established as important learning environment features within the context of higher education: Good Teaching (GT), Generic Skills (GS), Appropriate Assessment (AA), Appropriate Workload (AW), Clear Goals and Standards (CG), Emphasis on Independence (IN) [12, 14, 16]. Overall students indicated a preference for the BL compared to the OFC approach. We observed significant differences between the responses of the OFC and BL groups regarding the learning experience, perceived value of the flipped classroom, teaching process, skill development and the evaluation system outlined in Table 3. Due to small number of respondents in some categories, Strongly Agree and Agree, and additionally, Strongly Disagree and Disagree categories were joined for analysis. Furthermore, also relating to the small number respondents, the margin of error varied substantially, ranging from 5.7% to 12.6% for estimates of Agree/ Strongly Agree in the BL group, and 16.7% to 18.5% for estimates of Agree/ Strongly Agree in the OFC group.

**Table 3:** Comparison of students’ perspectives between online flipped classroom and blended learning.

STUDENTS	Agree (Strongly agree & agree)	Neutral	Disagree (Disagree & Strongly disagree)	p-value	Bonferroni corrected p-value
**Good Teaching scale**					
Q4. The teaching staff of this course motivate students to do their best work.					
OFC	64.29%	25.00%	10.71%	**<0.001**	**0.004**
BL	95.16%	4.84%	0.00%		
Q9. Staff here put a lot of time into commenting on students’ work.					
OFC	46.43%	21.43%	32.14%	**0.004**	0.159
BL	74.19%*	17.74%	8.06%		
Q20. The staff make a real effort to understand difficulties students may be having with their work.					
OFC	50.00%	17.86%	32.14%	**0.001**	**0.019**
BL	82.26%	14.52%	3.23%		
Q22. Teaching staff here normally give helpful feedback on how you are going.					
OFC	53.57%	17.86%	28.57%	0.066	0.999
BL	70.97%	17.74%	11.29%		
Q23. Our lecturers are extremely good at explaining things to us.					
OFC	71.43%	25.00%	3.57%	**0.05**	0.999
BL	88.71%	8.06%	3.22%		
Q25. Teaching staff here work hard to make subjects interesting.					
OFC	67.85%	17.86%	14.28%	**0.013**	0.463
BL	88.71%	9.68%	1.61%		
Q31. Staff show no real interest in what students have to say					
OFC	21.43%	17.86%	60.71%	0.067	0.999
BL	6.45%	16.13%	77.42%		
Q33. This course really tries to get the best out of all its students.					
OFC	42.85%	35.71%	21.42%	**0.001**	**0.033**
BL	79.03%	12.90%	8.06%		
**Clear Goals and Standards scale**					
Q1. It’s always easy here to know the standard of work expected.					
OFC	60.71%	17.86%	21.42%	0.088	0.999
BL	77.42%	12.90%	9.68%		
Q8. You usually have a clear idea of where you’re going and what’s expected of you.					
OFC	67.85%	10.71%	21.43%	**0.006**	0.226
BL	90.32%	6.45%	3.23%		
Q18. It’s often hard to discover what’s expected of you in this course.					
OFC	39.28%	14.29%	46.43%	0.19	0.999
BL	19.35%	25.81%	54.84%		
Q24. The aims and objectives of this course are NOT made very clear.					
OFC	21.43%	21.43%	57.15%	**0.027**	0.999
BL	8.06%	12.90%	79.04%		
Q35. The staff here make it clear right from the start what they expect from students.					
OFC	53.57%	25.00%	21.43%	0.389	0.999
BL	61.29%	25.81%	12.90%		
**Generic Skills scale**					
Q2. This course has helped me to develop my problem-solving skills.					
OFC	50.00%	25.00%	25.00%	**0.001**	**0.004**
BL	81.96%	18.03%	0.00%		
Q6. This course has sharpened my analytic skills.					
OFC	42.85%	25.00%	32.14%	**0.023**	0.858
BL	64.52%	24.19%	11.29%		
Q11. This course has helped develop my ability to work as a team member.					
OFC	25.00%	39.29%	35.71%	**<0.001**	**0.004**
BL	69.36%	19.35%	11.29%		
Q12. As a result of doing this course, 1 feel more confident about tackling unfamiliar problems.					
OFC	39.28%	39.29%	21.43%	**0.029**	0.999
BL	62.90%	27.42%	9.68%		
Q13. This course has improved my written communication skills.					
OFC	32.14%	14.29%	53.57%	0.222	0.999
BL	29.03%	41.94%	29.04%		
Q28. This course has helped me develop the ability to plan my own work.					
OFC	35.71%	25.00%	39.28%	**0.003**	0.107
BL	61.29%	30.65%	8.06%		
**Appropriate Assessment scale**					
Q7. Lecturers here frequently give the impression they have nothing to learn from students.					
OFC	35.71%	25.00%	39.28%	**0.001**	**0.048**
BL	3.28%	29.51%	67.21%		
Q10. To do well on this course all you really need is a good memory.					
OFC	39.28%	28.57%	32.14%	**0.007**	0.27
BL	14.52%	27.42%	58.07%		
Q17. Staff seem more interested in testing what you’ve memorised than what you’ve understood.					
OFC	32.14%	17.86%	50.00%	0.068	0.999
BL	14.52%	17.74%	67.75%		
Q26. Too many staff ask us questions just about facts.					
OFC	21.43%	21.43%	57.14%	0.785	0.999
BL	9.68%	35.48%	54.84%		
Q29. Feedback on student work is usually provided ONLY in the form of marks and grades.					
OFC	46.43%	21.43%	32.14%	0.364	0.999
BL	40.99%	13.11%	45.90%		
Q32. It would be possible to get through this course just by working hard around exam times.					
OFC	64.29%	10.71%	25.00%	0.146	0.999
BL	45.16%	20.97%	33.88%		
**Appropriate Workload scale**					
Q5. The workload is too heavy.					
OFC	0.00%	10.71%	89.28%	0.067	0.999
BL	8.06%	19.35%	72.58%		
Q14. It seems to me that the syllabus tries to cover too many topics.					
OFC	14.28%	10.71%	75.00%	0.2	0.999
BL	19.36%	20.97%	59.68%		
Q19. We are generally given enough time to understand the things we have to learn.					
OFC	53.57%	21.43%	25.00%	0.969	0.999
BL	56.45%	12.90%	30.65%		
Q27. There’s a lot of pressure on you as a student here.					
OFC	39.28%	35.71%	25.00%	**0.007**	0.274
BL	20.97%	22.58%	56.45%		
Q36. The sheer volume of work to be got through in this course means you can’t comprehend it all thoroughly.					
OFC	17.86%	21.43%	60.71%	0.536	0.999
BL	24.19%	20.97%	54.84%		
**Emphasis on Independence scale**					
Q3. There are few opportunities to choose the particular areas you want to study.					
OFC	42.86%	25.00%	32.14%	0.298	0.999
BL	46.77%	38.71%	14.52%		
Q15. The course has encouraged me to develop my own academic interests as far as possible.					
OFC	50.00%	28.57%	21.43%	0.095	0.999
BL	67.75%	20.97%	11.29%		
Q16. Students have a great deal of choice over how they are going to learn in this course.					
OFC	25.00%	17.86%	57.14%	**0.005**	0.185
BL	48.39%	27.42%	24.19%		
Q21. Students here are given a lot of choice in the work they have to do.					
OFC	21.43%	17.86%	60.71%	**0.031**	0.999
BL	35.48%	30.65%	33.88%		
Q30. We often discuss with our lecturers or tutors how we are going to learn in this course.					
OFC	25.00%	3.57%	71.43%	0.07	0.274
BL	35.48%	17.74%	46.78%		
Q34. There’s very little choice in this course in the ways you are assessed.					
OFC	89.28%	10.71%	0.00%	**0.004**	0.13
BL	59.68%	22.58%	17.75%		
Q37. Overall, I am satisfied with the quality of this course.					
OFC	82.15%	3.57%	14.28%	0.129	0.999
BL	91.94%	8.06%	0.00%		

#### Good Teaching scale

We observed that the BL delivery approach resulted in significantly greater levels of student satisfaction on the GT scale compared to the OFC approach. Specifically the BL group felt that the teaching staff motivated students to do their best (Q4, p<0.001), put a lot of time into commenting on students work (Q9, p = 0.004) and made a real effort to understand difficulties that students may be having with their work (Q20, p = 0.001). However, having adjusted for multiple comparisons Q9 was no longer significant.

Furthermore, compared to the OFC group the BL students felt that faculty were extremely good at explaining course content (Q23, p = 0.05) and that they made significant efforts to make the subjects interesting (Q25, p = 0.013). Critically we observed a significant improvement of student perceptions regarding the course trying to get the best out of its students among the BL group compared to the OFC student group (Q33, p = 0.001). However, having adjusted for multiple comparisons, only improvement of student perceptions regarding the course trying to get the best out of its students among the BL group compared to the OFC student group remained statistically significantly different (Q33, p = 0.033).

#### Clear Goals and Standards scale

There was no evidence of a difference in student perceptions on the goals and standards (CG) scale specifically about what was expected from them (Q18), about the standard of work required (Q1) and faculty expectations of students being made clear (Q35). Overall there was no evidence of a difference in goals and standards after Bonferroni adjustment, however, before adjustment there was some evidence the BL group were significantly more satisfied with the CG compared to the OFC. Specifically, students felt that they had a clear idea of what was going on and what was expected from them (Q8, p = 0.006) and that the aims and objectives of the course were made very clear (Q24, p = 0.027).

#### Generic Skills scale

There was no evidence of a difference in student perceptions about the capacity for the OFC or BL course to improve their written communication skills (Q13). In contrast to the OFC course students those who participated in the BL course showed some evidence of a difference prior to Bonferroni adjustment, finding it helped develop their problem skills (Q2, p = 0.001), sharpened their analytical skills (Q6, p = 0.023), developed their ability to work as a team member (Q11, p<0.001), improved their confidence about tackling unfamiliar problems (Q12, p = 0.029) and developed their ability to plan work (Q28, p = 0.003). Developing their problem skills (Q2) and developing their ability to work as a team member (Q11, remained statistically significant following adjustment for multiple comparisons.

#### Appropriate Assessment scale

There was no evidence of a difference between the OFC and BL group in student perceptions of the impression that staff are more interested in testing what students have memorised (Q17) or ask too many questions about facts (Q26). Additionally, there was no difference between the OFC and BL group in their perceptions of the form that feedback was given (Q29) or that just by working hard around exam times they could get through the course (Q32). We observed significantly greater levels of student satisfaction among the BL group with the impression that faculty can learn from students (Q7, p = 0.001) compared to the OFC group. Furthermore, the BL group indicated that doing well on the course required more than just a good memory (Q10, p = 0.007).

#### Appropriate Workload scale

There was no evidence of a difference in student perceptions in relation to the workload (Q5), the number of topics covered in the syllabus (Q14), the amount of time given to learn (Q19), the pressure felt by students (Q27) or how the volume of work affects comprehension of topics (Q36).

#### Emphasis on Independence scale

There was no evidence of a difference in student perceptions between the OFC and BL group on the IN scale regarding opportunities to choose the particular areas you want to study (Q3), that the course encouraged them to pursue their academic interests (Q15) or their opportunities to discuss how they were going to learn with lecturers (Q30). However, we observed that the BL group were significantly more satisfied with elements in the IN scale compared to the OFC group. Specifically, students in the BL group felt they had greater levels of choice regarding how they would learn (Q16, p = 0.005), the work they had to do (Q21, p = 0.031) and the ways in which they were assessed (Q34, p = 0.004).

#### Questions regarding the value of the flipped classroom

Previous studies have highlighted questions within the CEQ survey, which provide insights into the perceived value of the flipped classroom [21]. The FC scale questions overlap with the GT and GS scale; specifically questions 2, 4, 5, 11, 12, 13 and 28. Student survey responses indicated a significant level of student satisfaction with the online flipped classroom approach as part of the revised BL curriculum. As mentioned above students in the BL group felt that there were more opportunities to improve their problem-solving skills (Q2, P = 0.01) and that staff did more to motivate them (Q4, p<0.001). In addition, they felt the course helped develop their ability to work as a team member compared to the OFC group (Q11, <0.001), tackle unfamiliar problems (Q12, p = 0.029) and developed their ability to plan their own work (Q28, p = 0.003). When asked to rate the statement “Overall, I am satisfied with the quality of this course” there was no evidence of a difference in the rating between the OFC and the BL group (Q 37).

#### Comparison of overall student performance on final multiple-choice exam

Next we compared students’ exam scores before and after the educational intervention for all students in the OFC (n = 114) and BL groups (n = 257) and students in the OFC (n = 28) and BL groups (n = 59) who responded to the survey. Students answered 20 ophthalmology multiple-choice questions (MCQ) as part of completing the course. Each question had the same weight, and the total score was converted into a 0–100 scale. Independent samples t test was used to compare the differences between the two groups. This analysis of the final exam MCQ score showed that there were no statistical differences between the OFC and BL group (p = 0.0560). Comparison of the final exam MCQ score for survey responders between the OFC and BL found no evidence of a statistical difference in the score achieved. Overall, this indicates that the BL did not negatively influence knowledge gain.

## Discussion

The imperatives of the COVID-19 pandemic mandated an inevitable transition to online and distance learning, addressing the challenges posed by government directives and social distancing requirements. In the aftermath of the COVID-19 pandemic blended learning has become an accepted approach in health professions education [27]. While student safety and wellbeing was paramount, removing medical students from the clinical context to minimise risk associated with the COVID-19 pandemic was not a feasible long-term strategy. BL involves both face-to-face and online learning components and was therefore an advantageous approach to health professions education during the pandemic as it offers the best of both approaches [27]. In this study we wanted to assess student satisfaction with a revised ophthalmology module adopting a BL format, which included online learning and in-person seminars combined with practical patient centred sessions. Our goal was to compare BL with the previous delivery format that relied solely on a remote online flipped classroom to facilitate continued delivery of the ophthalmology module. It was hypothesised that as an educational intervention, the blended learning approach would continue to facilitate delivery of content as well as maintaining or improving levels of student satisfaction and knowledge gain as determined by a CEQ and MCQ examination.

Learner satisfaction is a multidimensional construct and is related to an individual’s subjective assessment [28]. Student satisfaction hinges on the efficacy of educational courses and the individual’s enthusiasm and enjoyment in the learning process [29]. Blended learning offers learners more choice in a multimodal delivery of course content, and when compared to traditional deliver, BL has been shown to yield more favourable results in terms of knowledge outcomes when compared to traditional learning in HPE [30]. While the analysis of the final exam MCQ scores in our study showed that there were no statistical differences between the OFC and BL groups, the BL group showed higher satisfaction with the choice provided by the BL approach with how they learn, the work they completed, and the methods of assessment (Emphasis on Independence scale). The practical constraints related to the OFC approach provided the learners fewer freedoms and choice in their educational journey.

Chick *et al* suggested innovative technology including FC could play an essential role bridging the educational gap during the unprecedented COVID-19 pandemic [31]. The FC has been demonstrated to be accessible and user friendly [32], and was favourable among ophthalmology residents, with reported improvement in test scores [33]. The OFC approach has been utilised by many academics who found it was well received by students and resulted in similar or enhanced knowledge gain in some instances compared to the traditional delivery of teaching [5–9]. However, our previous study’s findings were in contrast to this literature, and we found significant dissatisfaction with the online flipped classroom approach [10]. Given that our initial rapid response to the challenges of delivering content during the pandemic relied significantly on a remote OFC approach we sought to determine student perceptions with this model. Overall students reported a lack of satisfaction with this model indicating a lack of staff motivation, difficulties determining the standard of work required and lack of development of critical thinking and problem solving as issues with the OFC approach for remote ophthalmology teaching [10]. We believe that a lack of faculty preparedness [34–36], digital fatigue and student uncertainty may also have contributed to student dissatisfaction with the OFC approach [37–39]. In this study compared to the OFC group the BL students felt staff excelled at explaining course content (Q23, p = 0.05) and made significant efforts to make the subjects engaging (Q25, p = 0.013). The variety in the BL approach offers more choice and enables learners to engage with material through various mediums, and this was also reflected in the BL groups’ satisfaction with the choices regarding how they would learn and be assessed. Additionally, the multimodal delivery of course content in BL, appears to have addressed some of the challenges associated with the technological challenges faced by staff in the initial response to the pandemic.

Student Satisfaction is also associated with an individual’s interaction with their peers and with faculty [28], and in a national review in the UK having a “social life and meeting people” was acknowledged as a crucial factor contributing to overall satisfaction [40]. Our resulted demonstrated BL resulted in significantly greater levels of student satisfaction on the Good Teaching scale compared to the OFC approach, specifically relating to items associated with staff motivating students to do their best and to understand student difficulties. We noted markedly higher levels of student satisfaction within the BL group with survey items relating to students feeling motivated by staff (Q4), working as part of a team (Q11), and how the course tried to get the best of students (Q33). HPE involves hands on learning and elements of teamwork and effective communication. Online learning has been associated with poor engagement and during the COVID-19 pandemic, reduced interpersonal interaction [41], and has also been associated with lower levels of preparedness and a lack of hands-on training [42]. Our revised BL curricula, including online learning with in-person seminars and practical patient centred sessions, improved students self-reported problem solving, analytical skills and ability to work as part of team.

### Study limitations

The interpretations drawn from our investigations should be considered within the context of the limitations inherent to this study. One such limitation is the relatively low level of student engagement observed in these investigations, which subsequently led to suboptimal response rates and smaller sample sizes. This resulted in varying margins of error around estimates, and results should be interpreted with caution. The ongoing global pandemic during the participant recruitment phase represents a factor potentially influencing the lack of study participants.

Furthermore, our study encompasses participants from two distinct iterations of clinical attachments spanning across two academic years. It is noteworthy, however, that an analysis of each student cohort, as well as those who actively engaged in the study, determined no statistically significant disparities in terms of characteristics/demographics.

## Conclusion

The COVID-19 pandemic compelled an inevitable shift to online and distance learning to address challenges posed by government mandates and social distancing requirements. However, in post-pandemic HPE, it is crucial to assess the effectiveness and learner perceptions of online and distance learning interventions. In line with recent BEME reviews we implemented a revised curriculum which included a blend of traditional classroom-based and remote learning approaches combined with in person practical elements including direct patient contact with mitigated risk. We provided support and training of both faculty and students will help to increase digital proficiency and engagement as online elements are continuing as a central feature of medical education. These changes resulted in significant increases in student satisfaction. Our study revealed a substantial student preference for blended learning (BL) over the online flipped classroom (OFC) approach, with comparable student performances based on MCQ examinations. Importantly, this study presents a unique insight into the repercussions of introducing an educational intervention centred on blended learning amidst the pandemic. This insight focusses on student satisfaction and the enhancement of learning experiences, underlining the distinctive value of our research. These findings indicate a preference for reintegrating in-person and patient engagement activities in post-pandemic health professions education.

## Supporting information

S1 Appendix Ophthalmology module learning outcomes. (DOCX)
